# Geometric and Dosimetric Evaluation of Deep Learning-Based Automatic Delineation on CBCT-Synthesized CT and Planning CT for Breast Cancer Adaptive Radiotherapy: A Multi-Institutional Study

**DOI:** 10.3389/fonc.2021.725507

**Published:** 2021-11-09

**Authors:** Zhenhui Dai, Yiwen Zhang, Lin Zhu, Junwen Tan, Geng Yang, Bailin Zhang, Chunya Cai, Huaizhi Jin, Haoyu Meng, Xiang Tan, Wanwei Jian, Wei Yang, Xuetao Wang

**Affiliations:** ^1^ Department of Radiation Therapy, The Second Affiliated Hospital, Guangzhou University of Chinese Medicine, Guangzhou, China; ^2^ School of Biomedical Engineering, Southern Medical University, Guangzhou, China; ^3^ Department of Oncology, The Fourth Affiliated Hospital, Guangxi Medical University, Liuzhou, China

**Keywords:** deep learning, automatic delineation, synthetic CT, dosimetric evaluation, adaptive radiotherapy

## Abstract

**Purpose:**

We developed a deep learning model to achieve automatic multitarget delineation on planning CT (pCT) and synthetic CT (sCT) images generated from cone-beam CT (CBCT) images. The geometric and dosimetric impact of the model was evaluated for breast cancer adaptive radiation therapy.

**Methods:**

We retrospectively analyzed 1,127 patients treated with radiotherapy after breast-conserving surgery from two medical institutions. The CBCT images for patient setup acquired utilizing breath-hold guided by optical surface monitoring system were used to generate sCT with a generative adversarial network. Organs at risk (OARs), clinical target volume (CTV), and tumor bed (TB) were delineated automatically with a 3D U-Net model on pCT and sCT images. The geometric accuracy of the model was evaluated with metrics, including Dice similarity coefficient (DSC) and 95% Hausdorff distance (HD95). Dosimetric evaluation was performed by quick dose recalculation on sCT images relying on gamma analysis and dose-volume histogram (DVH) parameters. The relationship between ΔD95, ΔV95 and DSC-CTV was assessed to quantify the clinical impact of the geometric changes of CTV.

**Results:**

The ranges of DSC and HD95 were 0.73–0.97 and 2.22–9.36 mm for pCT, 0.63–0.95 and 2.30–19.57 mm for sCT from institution A, 0.70–0.97 and 2.10–11.43 mm for pCT from institution B, respectively. The quality of sCT was excellent with an average mean absolute error (MAE) of 71.58 ± 8.78 HU. The mean gamma pass rate (3%/3 mm criterion) was 91.46 ± 4.63%. DSC-CTV down to 0.65 accounted for a variation of more than 6% of V95 and 3 Gy of D95. DSC-CTV up to 0.80 accounted for a variation of less than 4% of V95 and 2 Gy of D95. The mean ΔD90/ΔD95 of CTV and TB were less than 2Gy/4Gy, 4Gy/5Gy for all the patients. The cardiac dose difference in left breast cancer cases was larger than that in right breast cancer cases.

**Conclusions:**

The accurate multitarget delineation is achievable on pCT and sCT *via* deep learning. The results show that dose distribution needs to be considered to evaluate the clinical impact of geometric variations during breast cancer radiotherapy.

## Introduction

Intensity-modulated radiotherapy (IMRT) after breast-conserving surgery significantly improves the survival of breast cancer patients (1). However, there are the patient setup error and anatomical structure changes during the interfractional radiotherapy (2). The variation range of mean central lung distance is 0.59–2.94 cm (3). The mean 3D displacement of patient setup is 7.3 and 7.6 mm by laser and port film setup, respectively (4). The deviation could lead to inconsistencies between the actual delivery dose and the planning dose (5). Large interfraction variation is observed, motivating the need for adaptive radiotherapy. Adaptive radiotherapy can automatically adjust the plan according to changes in the target volume (6, 7). When the patient is lying on the couch waiting for treatment, plan evaluation and adaptation need to be completed as quickly as possible. Online adaptation, which requires real-time delineation of the contours of the target volumes and organs at risk (OARs) for re-planning, is a promising technique (8). Some studies have been conducted for online adaptation, especially for prostate cancer as well as for head and neck cancer (9–13). Cone-beam CT (CBCT) is a common tool for location verification in radiotherapy and can be used for plan adaptation (14, 15). However, imaging artifacts caused by respiratory movement make CBCT-based adaptive radiotherapy for breast cancer infeasible. CBCT images cannot be directly used for dose calculation due to inaccurate HU values and needs to be converted into synthetic CT for dosimetric evaluation (16–19).

The delineation of target volumes and OARs is a prerequisite for adaptive radiotherapy. However, manual delineation is time-consuming and labor-intensive and cannot meet the requirements of real-time adaptive radiotherapy (20). It is necessary to build an automatic delineation model (21, 22). Some researchers used atlas-based segmentation software for delineation of target volumes on computed tomography (CT) images for radiotherapy. Dice score of segmentations with these commercial software is not high enough (23–26). CBCT-based delineation can be achieved by deformable image registration and direct delineation on CBCT images. Deformable image registration could transfer the contours to CBCT images from planning CT images (27). However, deformable registration relying on the image quality and algorithm cannot perform well for patients with large variations, leading to uncertainty in propagating contours (28). Direct CBCT-based delineation can reduce uncertainty from registration errors. Schreier et al. (29) investigated segmentation for the male pelvis using CBCT and CT images. Peroni et al. (11) developed an automatic strategy to generate online virtual CT and automatically segmented structures using CBCT and virtual CT images for head and neck cancer adaptive radiation therapy. Inter-observer variability is high in the delineation of target volumes and OARs on CT and CBCT scans of the chest. At present, most of the studies on the automatic segmentation of chest medical images do not perform well, and it is necessary to develop a model with better performance to delineate all the target volumes and OARs accurately at one time. Additionally, the geometric metrics do not fully indicate clinical quality. Therefore, it is necessary to evaluate the performance of the automatic delineation model in terms of clinical applicability (30). The geometric and dosimetric changes between planning CT (pCT) and synthetic CT (sCT) needs to evaluated due to high clinical significance for adaptive therapy.

In our study, we investigated the feasibility of automatically delineating multiple contours based on deep learning for breast cancer radiation therapy. The synthetic CT image was first generated from CBCT images with a cycle generative adversarial network (cycleGAN). Second, we developed an automatic delineation model using 3D U-net based on pCT and the radiotherapy structure of breast cancer patients to delineate the target volumes and OARs on planning CT and synthetic CT images, respectively. Third, the treatment plan was transferred to the synthetic CT image from the planning CT image. It could be verified quantitatively by quick dose recalculation for dosimetric evaluation. The Flowchart of the proposed method is shown as **Supplementary Figure 1** in the **Supplementary Material**. The clinical impact of geometric variations in target volumes and OARs was evaluated to provide the feasibility for breast cancer adaptive radiotherapy.

## Materials and Methods

### Patient Datasets

Datasets obtained from two medical institutions in China between January 2014 and December 2020 were analyzed retrospectively. A total of 1,127 patients (institution A: 1,074/institution B: 53) who received radiotherapy after breast-conserving surgery were included. The data of 75 patients from institution A including pCT and CBCT images with BH were split into 52 samples for training and 23 samples for testing on CBCT-to-CT synthesis. The pCT images and structures of 1,052 patients were randomly divided into a training set (700 patients), validation set (100 patients), and test set (252 patients) for automatic delineation model training and evaluation. Among the test set, 199 patients were from institution A, and 53 patients were from institution B. The patient characteristics are summarized in **Table 1**.

**Table 1:** Summary of patient characteristics.

Patient characteristics	Value
Age range (y)	22–72
Laterality	
Right	600
Left	527
Institution	
A	1,074
B	53
Stage, No.	
0	92
I	530
II	433
III	72

Patients were immobilized on the breast bracket before obtaining a CT scan with 5 mm slices (SOMATOM Sensation Open, Siemens). The dimensions of the images were 512 × 512 voxels for each slice. CBCT is widely used for target position verification and setup error correction during breast patient radiation therapy. In this study, CBCT images were captured by a Varian Edge treatment machine (Varian Medical Systems, Palo Alto) in half-fan mode utilizing breath-hold (BH) guided by AlignRT (Vision RT Ltd, London, UK). The images with 512 × 512 voxels for each slice were reconstructed with a 1.99 mm slice thickness. The quality of CBCT with the BH technique is much higher than that with conventional scan. CBCT could be used to generate accurate synthetic CT for dose recalculation.

Clinical target volumes (CTV), tumor bed (TB), and organs at risk (OARs) were delineated by two radiation oncologists according to the ESTRO consensus guideline. The director with 20 years’ experience of the radiation oncology department of the corresponding institution was consulted in cases of disagreement. Manually delineated contours were used as ground truth for training and testing. The radiotherapy plans were designed with the Pinnacle3 treatment planning system (Philips Radiation Oncology Systems, Philips Healthcare) by combining 3D conformal radiation therapy (CRT) and intensity modulated radiation therapy (IMRT). The prescribed dose was 52.2 Gy given to CTV and 63.8 Gy given to TB in 29 fractions. CRT achieved 80% of the total dose, and IMRT achieved 20% of the total dose. One hundred percent of the prescribed dose covered 95% of the volume of the target area. The physicist first designed the plan, and then the radiation oncologist and the physicist jointly evaluated the plan before implementation to ensure the quality of the plan.

### Automatic Delineation of Target Volumes and Organs at Risk

**Figure 1:**
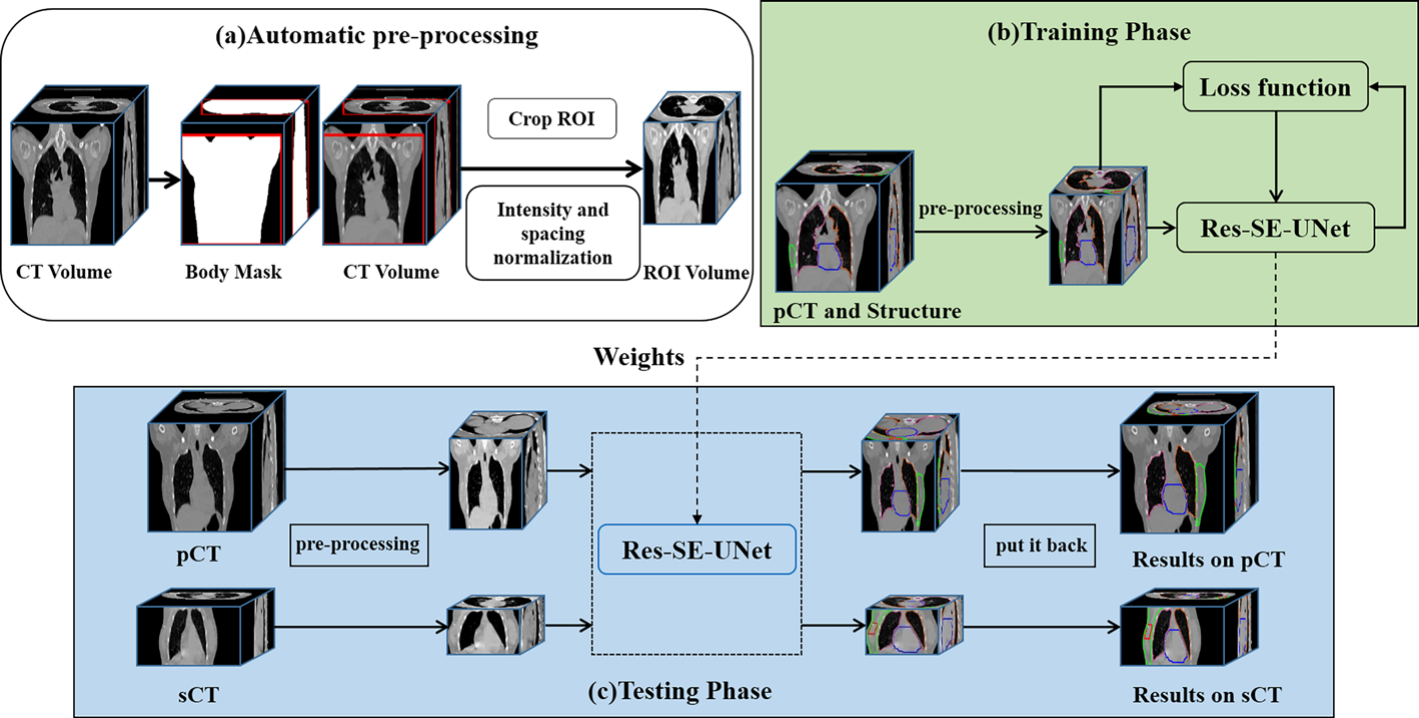
The workflow of the automatic delineation model. **(A)** automatic preprocessing, **(B)** training phase, **(C)** testing phase.

Our automatic delineation model is applied to 3D volume. Patch-based training makes the model cannot judge left and right lungs and target volumes. Therefore, through a series of preprocessing steps, as shown in **Figure 1A**, we feed the human body volume as completely as possible into the network, so that the network has a larger receptive field. First, the Hough transform line detection, the threshold method, and the morphological method were used to remove the bed and obtain a human body mask. Then, the minimum circumscribed cube is cropped from the body mask, which is the region of interest (ROI) of each subject. Finally, the spatial resolution of all ROIs is converted to 2 × 2 × 5 mm^3^, and the intensity is normalized by z-score for training.

We use Res-SE-U-Net (31) as the automatic delineation network, which is a modified 3D U-Net (32). Res-SE-U-Net includes the down-sampling path, up-sampling path, and skip-connection layer, which can extract the multiscale features of images. In addition, the addition of Res-block and SE-block leads to its stronger feature extraction ability than the original U-Net. The workflow of the automatic delineation model is shown in **Figure 1**. Training of a network took about 48 h, whereas all the target volumes and OARs for one patient were predicted in 5 s.

### CBCT-to-CT Synthesis

We use a 2D cycleGAN (33), which is an unsupervised image-to-image translation deep learning framework, to generate sCT. The cycleGAN contains two generators (*G_CBCT_*_–_ *_CT_* and *G_CT–CBCT_*) and two discriminators (*D_CT_* and *D_CBCT_*). The generator *G_CBCT_*_–_ *_CT_* takes CBCT as input and generates the sCT; in contrast, *G_CT–CBCT_* takes CT as input and generates the synthetic CBCT. The discriminator *D_CT_* and *D_CBCT_* discriminates whether the CT or CBCT images are real or synthesized, respectively. The sCT of one patient could be generated in 3–4 s by the trained model. The schematic flow is shown as **Supplementary Figure 2** in the **Supplementary Material**.

The optimization of the cycleGAN includes two objective functions: adversarial loss and cycle consistency loss. The antagonistic objectives of generators and discriminators are reflected in adversarial loss. We denote the data distribution as *I_CT_* ~ *p*_data_(*I_CT_*) and *I_CBCT_* ~ *p*_data_(*I_CBCT_*). The adversarial loss is expressed as:


(1) $$\begin{array}{c} {ℒ}_{GAN}\left(G_{CBCT-CT},D_{CT},I_{CT},I_{CBCT}\right)=E_{I_{CT}\sim p_{data\;}(I_{CT})}\left[\log D_{CT}(I_{CT})\right]\\ +E_{I_{CBCT}\sim p_{data\;}(I_{CBCT})}\left[\log\left(1-D_{CT}(G_{CBCT-CT}(I_{CBCT}))\right)\right] \end{array}$$

where *I_CBCT_* is the real CBCT and *G_CBCT–CT_* (*I_CBCT_*) is the synthetic CT generated by *G_CBCT–CT_*.

### Geometric Evaluation

#### Automatic Delineation Performance Evaluation

The performance of the automatic delineation model was evaluated on pCT and sCT, respectively. To quantitatively assess the delineation accuracy, we used two metrics: Dice similarity coefficient (DSC) and 95% Hausdorff distance (HD95). DSC describes the spatial overlap between the automated delineation and the ground-truth. The metrics HD95 was used to evaluate the shape difference in the study. The equations are defined as **Supplementary Equations** (1, 2) in the **Supplementary Material**.

#### CBCT-Synthesized sCT Quality Evaluation

Mean error (ME), mean absolute error (MAE), peak signal to noise ratio (PSNR), structural similarity index (SSIM), and spatial non-uniformity (SNU) were used to evaluate the image quality of sCT and CBCT, respectively. The formulas for these metrics are defined as **Supplementary Equations (4–8)** in the **Supplementary Material**. We selected five regions of interest (ROIs) to calculate the SNU, as shown in **Supplementary Figure 3** of the **Supplementary Material**. ME and MAE are the magnitudes of the difference between the pCT and the sCT. The lower these values are, the better the image quality is. High PSNR and SSIM mean high image quality. In this study, deformable registration was performed on the sCT to align it with pCT, and the metrics were calculated within the body mask of the sCT.

### Dosimetric Evaluation

Dosimetric accuracy was evaluated based on the sCT images using clinical breast cancer treatment plans. A quick dose recalculation on the sCT images was performed to verify the treatment plan. The treatment plan that was transferred to the sCT from the pCT kept the same parameters as the original pCT-based plan. The difference in dose distribution between pCT and sCT was evaluated with gamma analysis and dose-volume histogram (DVH) parameters. The difference in DVH metrics of target volumes and OARs between pCT and sCT were also assessed for quantitative dosimetric evaluation. The DVH metrics of the target volumes, including D90, D95, and V95, were analyzed. Target coverage was defined as the dose received by 90 and 95% of the target volume (D90, D95) and the percent volume receiving 95% of the prescribed dose (V95) for the TB and CTV. If the dose difference in the target volumes and OARs on the sCT exceeds the threshold, it needs to rescan the pCT for re-planning. Δ*D*90, Δ*D*95, and Δ*V*95 are defined as:


(2) ΔD90=|D90(pCT)−D90(sCT)|

(3) ΔD95=|D95(pCT)−D95(sCT)|

(4) ΔV95=|V95(pCT)−V95(sCT)|

We investigated the relationship between the DSC and the dose difference to evaluate the effect of anatomical changes on dose during radiotherapy. sCT images were rigidly registered to pCT by reference to the bony landmarks. DSC-CTV for automatically delineating CTV on sCT images compared with manually delineating target volumes on pCT images was correlated with dosimetric metrics.

### Clinical Evaluation

The reproducibility and robustness of the automatic delineation model were evaluated using DSC and HD95 in a multi-institution study. The training set was from institution A. Of the 252 patients in the test set, 199 were from institution A and 53 were from institution B. The robustness of the model was validated by multi-institutional testing.

Clinical evaluation of the automatic delineation model was performed on an independent test set of 199 pCT scans and 23 sCT from institution A. The automatically delineating contours were checked by three groups a, b, and c in institution A based on their clinical experience. Each group consisted of two radiation oncologists. Three groups who were blinded to the ground truth contours reviewed the automatic delineating contours. The evaluation results were acceptable with no corrections, acceptable with minor corrections, and unacceptable respectively. Finally, the acceptable ratio of all the targets and OARs was analyzed.

### Statistical Analysis

MATLAB (version 2018b, The MathWorks Inc, Natick, MA, USA) was used for statistical analysis. A t test was used to test the statistical significance of the absolute difference of the dosimetric metrics for both plans, and a Spearman’s rank correlation test was used for correlation testing between DSC and ΔV95 or ΔD95 of CTV. Two-sided p-values were provided, and p-values <0.05 were considered significant.

## Results

### Geometric Evaluation

The ME, MAE, PSNR, and SSIM comparisons between the CBCT, synthetic CT (sCT), and planning CT (pCT) images are shown in **Table 2**. The average ME and MAE between CBCT and pCT images within the body was −37.71 ± 15.49 and 86.42 ± 10.12 HU, whereas the average ME and MAE between sCT and pCT images was 8.46 ± 11.88 and 71.58 ± 8.78 HU. The mean SNUs for CBCT, sCT, and pCT were 9.22 ± 3.89, 4.95 ± 4.13, 2.12 ± 0.85%, respectively. The HU value of sCT image is much closer to that of pCT image than that of CBCT image. The similarity increased obviously between sCT and pCT images with lower ME, MAE and higher PSNR, SSIM compared to CBCT and pCT images. The detailed comparison between pCT and sCT is shown as **Supplementary Figure 3** in the **Supplementary Material**.

**Table 2:** Similarity analysis between CBCT and pCT images, sCT and pCT images with all the testing patient datasets.

Type	ME (HU)	MAE (HU)	PSNR (dB)	SSIM	SNU (%)
CBCT *vs* pCT	−37.71 ± 15.49	86.42 ± 10.12	20.19 ± 5.26	0.88 ± 0.04	9.22 ± 3.89 *vs* 2.12 ± 0.85
sCT *vs* pCT	8.46 ± 11.88	71.58 ± 8.78	23.34 ± 3.63	0.92 ± 0.02	4.95 ± 4.13 *vs* 2.12 ± 0.85

**Figure 2:**
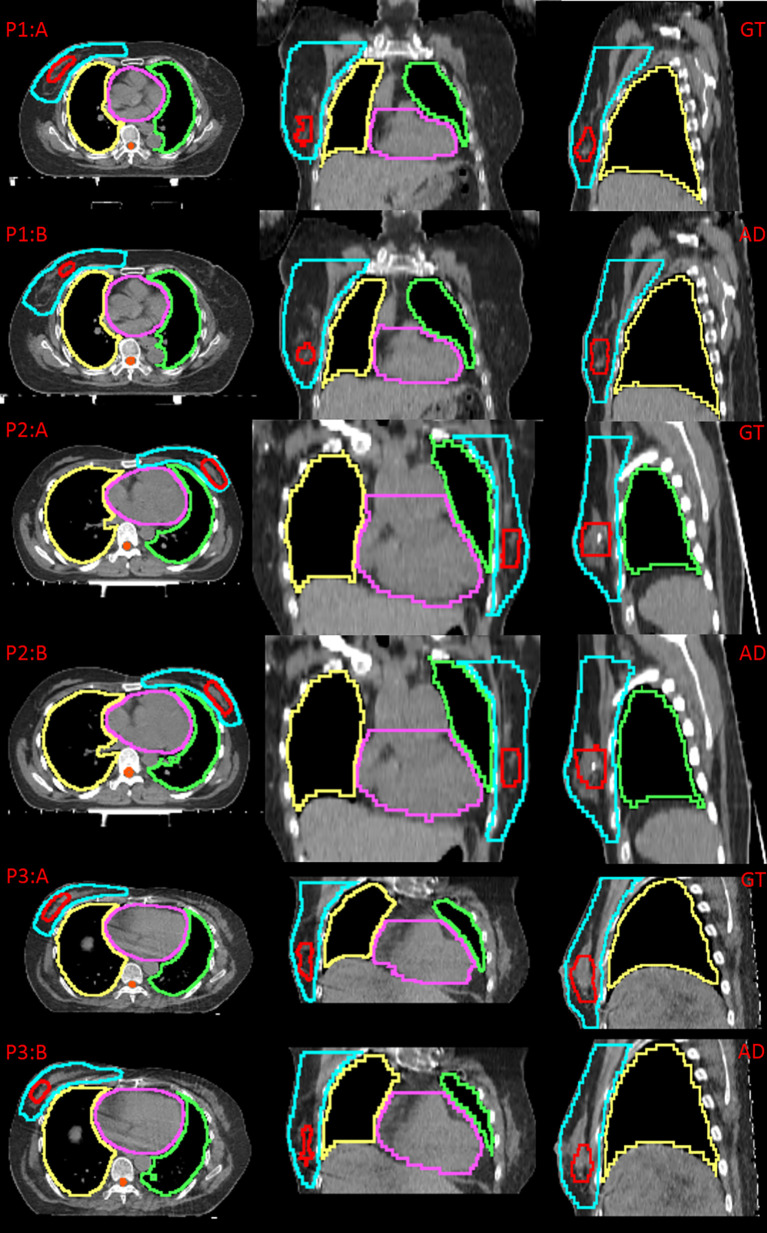
Comparison between the ground truth (GT) and automatic delineation (AD) at axial plane, coronal plane, sagittal plane, P1:A indicates GT on pCT for patient 1, P1:B indicates AD on pCT for patient 1; P2:A indicates GT on pCT for patient 2, P2:B indicates AD on pCT for patient 2; P3:A indicates GT on sCT for patient 3, P3:B indicates AD on sCT for patient 3.

**Figure 2** shows examples of the ground truth and the contours of automatic delineation on pCT and sCT images. There is good consistency for CTV and OARs between automatic delineation and manual delineation from human experts on pCT. The concordance can be found to decrease at the upper and lower bounds of the CTV from 2D sagittal sCT images. The automatically drawn tumor bed (TB) on sCT is obviously larger than the manually drawn TB.

The testing results of automatic delineation of multiple institutions are shown in **Table 3**. The results of pCT were calculated among 199 patients from institution A and 53 patients from institution B, respectively. The results of sCT were calculated among 23 patients from institution A for CBCT-to-CT synthesis testing cohorts. Good DSC and HD95 scores were found for the most contours on pCT (DSC: 0.73–0.97, HD95: 2.22–9.36 mm). The performance was slightly lower for the contours on sCT from institution A (DSC: 0.63–0.95, HD95: 2.30–19.57 mm). The mean DSC of CTV was 0.88 ± 0.03 for pCT, and 0.83 ± 0.03 for sCT, respectively. The segmentation model was also effective for pCT from institution B (DSC: 0.70–0.97, HD95: 2.10–11.43 mm). The mean DSC of CTV on pCT from institution B was 0.80 ± 0.06. The accuracy of automatic delineation for the datasets from institution B was lower than that from institution A.

**Table 3:** Quantitative results (Mean ± SD) of automatic delineation performance in multiple institutions.

Institution	Image	Metrics	TB	CTV	Heart	Left lung	Right lung	Spinal cord
A	pCT	DSC	0.73 ± 0.08	0.88 ± 0.03	0.93 ± 0.06	0.97 ± 0.01	0.97 ± 0.01	0.82 ± 0.05
		HD95/mm	9.36 ± 4.80	9.13 ± 4.04	7.63 ± 5.60	2.22 ± 1.37	2.59 ± 2.24	5.12 ± 5.90
	sCT	DSC	0.63 ± 0.08	0.83 ± 0.03	0.90 ± 0.02	0.94 ± 0.01	0.95 ± 0.01	0.81 ± 0.03
		HD95/mm	19.57 ± 17.01	10.81 ± 4.81	9.31 ± 2.60	5.75 ± 1.61	5.62 ± 1.72	2.30 ± 0.32
B	pCT	DSC	0.70 ± 0.09	0.80 ± 0.06	0.92 ± 0.02	0.97 ± 0.01	0.97 ± 0.00	0.73 ± 0.07
		HD95/mm	11.43 ± 6.17	18.22 ± 6.94	8.51 ± 3.71	2.10 ± 0.54	2.45 ± 1.00	8.38 ± 5.90

The clinical evaluation showed that the acceptable ratios of OARs, CTV, and TB were 76.38–100, 70.35–83.92, and 53.27–57.79% on pCT images and 73.91–82.61, 69.57–78.26, and 43.48–52.17% on sCT images, respectively, as shown in **Table 4**. Overall, the automatic delineation of CTV and OARs was clinically acceptable after minor corrections by the evaluation of medical group a, b, and c.

**Table 4:** Acceptable ratio for automatic delineation among different groups.

Image	Group	Acceptable ratio/%					
		CTV	TB	Left lung	Right lung	Heart	Spinal cord
pCT	a	76.88	57.79	100	98.99	90.95	81.91
	b	83.92	54.77	100	98.49	76.88	79.40
	c	70.35	53.27	95.98	96.48	88.44	76.38
sCT	a	73.91	52.17	95.65	100	86.96	82.61
	b	78.26	47.83	91.30	95.65	73.91	78.26
	c	69.57	43.48	86.96	82.61	82.61	73.91

### Dosimetric Evaluation

**Figure 3:**
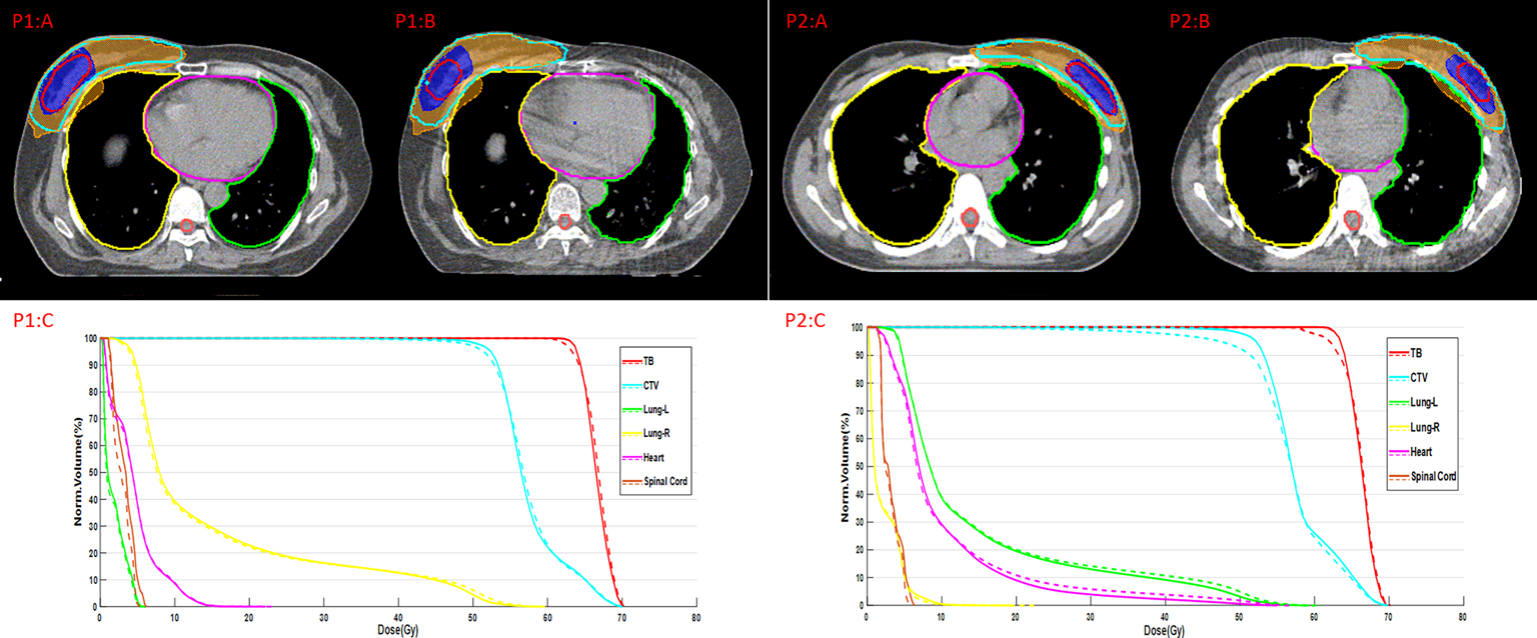
Dose distribution and DVH of the plans on pCT and sCT.

The dose distribution and dose-volume histogram (DVH) of the plan on synthetic CT (sCT) and planning CT (pCT) are shown in **Figure 3**. P1:A and P2:A indicate the dose distribution on pCT, and P1:B and P2:B indicate the dose distribution on sCT. The red line represents TB, and the blue shaded area represents the dose of 63.8 Gy. The wathet line represents CTV, and the yellow shaded area represents the dose of 52.2 Gy. P1:C and P2:C indicate the DVH of the two plans. The solid line represents the DVH on pCT, and the dotted line represents the DVH on sCT.

**Figure 4:**
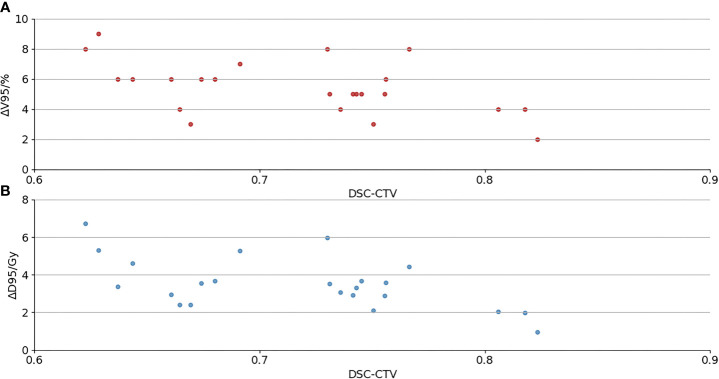
**(A)** The relationship between DSC and ΔV95 of CTV; **(B)** the relationship between DSC and ΔD95 of CTV.

Within the body, the mean ± standard deviation with 2%/2 mm and 3%/3 mm pass rates for the sCT images were 85.09 ± 6.28 and 91.46 ± 4.63% respectively. There was a negative correlation between DSC and ΔV95 or ΔD95 (r= −0.52, p= 2.4075E-11 and r= −0.51, p= 4.5815E-11, respectively). DSC of CTV down to 0.65 accounted for a variation of more than 6% of V95 and 3 Gy of D95 for CTV. DSC of CTV up to 0.80 accounted for a variation of less than 4% of V95 and 2 Gy of D95 for CTV, as shown in **Figure 4**.

Dosimetry evaluation of the plans on sCT *versus* original plans for 29 fractions is summarized in **Table 5**. The mean ΔV95 of CTV is less than 6%. The mean ΔD90/ΔD95 of CTV is less than 2 Gy/4 Gy. The mean ΔD90/ΔD95 of tumor bed was less than 4 Gy/5 Gy for all patients. We observed an absolute difference of more than 8% of the ΔV95 of TB. The poor delineation accuracy of TB leads to large dosimetry errors. The mean ΔD95 of CTV and ΔV10 of the heart are 4.20 ± 1.45 Gy and 3.92 ± 3.29% in the left-sided patients, 2.84 ± 0.84 Gy and 1.60 ± 1.96% in the right-sided patients, respectively. The dosimetric difference of target volume and heart in left-sided patients is greater than that in right-sided patients. P-values of the dosimetric difference of the TB and CTV were below 0.05, and p-values of the dosimetric difference of the OAR were over 0.05.

**Table 5:** Absolute difference of the dosimetric metrics for both plans of 23 patients.

Structure	Metrics	Absolute difference (Mean ± SD)			p-value
		Right breast	Left breast	All patients	
Spinal Cord	ΔDmax[Gy]	0.35 ± 0.28	0.34 ± 0.57	0.34 ± 0.45	0.63
Ipsilateral Lung	ΔV20[%]	1.80 ± 1.55	1.83 ± 1.59	1.82 ± 1.53	0.87
	ΔDmean[Gy]	0.83 ± 0.75	1.03 ± 1.02	0.94 ± 0.89	0.34
Heart	ΔV10[%]	1.60 ± 1.96	3.92 ± 3.29	2.86 ± 2.95	0.91
	ΔV30[%]	0.10 ± 0.32	2.58 ± 3.00	1.45 ± 2.52	0.56
	ΔDmean[Gy]	0.19 ± 0.19	1.72 ± 2.17	1.03 ± 1.76	0.96
TB	ΔD90[Gy]	2.65 ± 1.35	4.33 ± 2.54	3.57 ± 2.21	8.18E-09
	ΔD95[Gy]	3.73 ± 1.62	5.58 ± 2.50	4.74 ± 2.30	2.62E-07
	ΔV95[%]	5.70 ± 3.56	10.25 ± 7.03	8.18 ± 6.06	1.32E-07
CTV	ΔD90[Gy]	1.68 ± 0.57	1.89 ± 0.65	1.80 ± 0.61	2.61E-13
	ΔD95[Gy]	2.84 ± 0.84	4.20 ± 1.45	3.58 ± 1.37	2.30E-12
	ΔV95[%]	4.70 ± 1.25	6.17 ± 1.95	5.50 ± 1.79	1.01E-15

## Discussion

Adaptive radiotherapy based on CBCT for patient setup is a promising approach for improving treatment accuracy (34). Liu et al. (35) developed a deep learning approach to generate CBCT-based synthetic CT images and validated the dose calculation accuracy for clinical use in CBCT-guided pancreatic adaptive radiotherapy. However, sCT-based segmentation was not involved, which was also the key factor in adaptive radiotherapy. In our study, the synthetic CT image quality analysis of the thorax yielded an ME/MAE of 8.46/71.58 HU, and our results were better than those reported by Eckl et al. (36), with 29.6/94.2 HU. The SNU in synthetic CT was close to the SNU in planning CT. It demonstrated that synthetic CT had enough quality for contour delineation and dose calculation. The automatic delineation model that was developed by 3D Res-SE-U-Net based on the planning CT and RT structures made full use of the 3D correlative information between image slices. The automatic delineation model performs well enough with 0.88 DSC for CTV on planning CT. The performance of the model was lower for synthetic CT because of the inaccurate HU and image artifacts caused by respiratory in synthetic CT. The clinically meaningful evaluation of the performance of the model should include not only geometric difference but also dosimetric assessment (37, 38). In this study, the geometric and dosimetric differences between the contours on planning CT and the contours on synthetic CT were analyzed to assess the clinical impact of the changes in target volumes and OARs during radiotherapy. The interobserver variability is large for the contours of breast cancer, resulting in difficulty delineating exactly (39). We improved the robustness of the model by enlarging the datasets to over 1,000. The automatic delineation of CTV and OARs was more consistent with the manual delineation due to their regular shapes and locations. Tumor bed was significantly different between automatic and manual contours because the position of the tumor bed varied greatly for each patient. The results of the multi-institutional test showed that the model is robust and accurate. The performance of the model on the datasets from institution B was worse than that from institution A. Our approach was effective in dosimetric verification based on synthetic CT from CBCT, and ΔV95 and ΔD95 of CTV could be used as dosimetric metrics for rescanning pCT. There was a correlation between DSC and ΔD95 and ΔV95 for CTV; however, it was not an inversely proportional relationship. Poor DSC scores do not necessarily lead to inferior CTV dosimetry. The dose variation between the automatic delineation CTV on sCT images and the manual delineation CTV on planning CT images was not large if the DSC value was low due to the automatic delineation being too small. We evaluated the absolute difference of the dosimetric metrics for both plans. Geometric changes on synthetic CT have a greater impact on the cardiac dose difference in left breast cancer, and special attention needs to be paid to assess the cardiac dose for left breast cancer. ΔV95, ΔD95, and ΔD90 could be used as evaluation indicators for whether to re-plan.

Additional limitations include the following: (1) Deformable registration from planning CT to CBCT was performed because of the different slice thicknesses and scanned areas between the two images. Although deformable registration was used, it was difficult to align the anatomical structure in CBCT with the same structure in planning CT. The image quality of synthetic CT could be degraded due to the registration errors, which affect the delineation accuracy of the model to a certain extent. (2) DSC-CTV was computed by rigid registration between synthetic CT and planning CT. However, the limited registration accuracy could cause certain dosimetric uncertainties in CTV. (3) The synthetic CT was generated from any day’s CBCT, not entire treatment course. The anatomical changes and dosimetric difference were not evaluated during entire treatment delivery course.

The ranges of clinically acceptable ratio for CTV delineation are between 70% and 83% among the different groups, showing no common objective evaluation of the delineation. Variability exists between observer groups, demonstrating that the difference between automatic and manual delineation depends not only on contouring routines and guidelines, but also on personal preference. In the future, we hope to develop a universal model that can not only meet the quality requirements of multiple clinical institutions, but also adapt to the personal preferences of each observer. The automatically generated contours could be carefully reviewed by the radiation oncologist and used for treatment planning.

## Conclusion

This study demonstrated that the developed approaches are capable of reliably generating target and OAR contours on pCT and daily sCT images from CBCT images, which could greatly accelerate the re-planning process and meet the requirements of online plan adaptation. The automatic delineation model performed sufficiently well for most patients. The geometric and dosimetric differences between pCT and sCT images in fractional radiotherapy need to be evaluated due to the high clinical significance for breast cancer adaptive radiotherapy.

## Data Availability Statement

The raw data supporting the conclusions of this article will be made available by the authors, without undue reservation.

## Ethics Statement

The studies involving human participants were reviewed and approved by the medical ethics committee of the Second Affiliated Hospital of Guangzhou University of Chinese Medicine. Written informed consent for participation was not required for this study in accordance with the national legislation and the institutional requirements.

## Author Contributions

ZD and YZ: design of methodology, development and implement of models, original drafting. LZ: data curation and preprocessing. GY, BZ, CC, and WJ: experimental results analysis, draft reviewing. JT, HJ, HM, and XT: data collection. WY and XW: design of methodology, review and editing. All authors contributed to the article and approved the submitted version.

## Funding

This work is supported in part by the National Natural Science Foundation of China (81771916) and Guangzhou Science and Technology Plan (202102010264).

## Conflict of Interest

The authors declare that the research was conducted in the absence of any commercial or financial relationships that could be construed as a potential conflict of interest.

## Publisher’s Note

All claims expressed in this article are solely those of the authors and do not necessarily represent those of their affiliated organizations, or those of the publisher, the editors and the reviewers. Any product that may be evaluated in this article, or claim that may be made by its manufacturer, is not guaranteed or endorsed by the publisher.
