# Supplementary material for: Geometric and Dosimetric Evaluation of Deep Learning-Based Automatic Delineation on CBCT-Synthesized CT and Planning CT for Breast Cancer Adaptive Radiotherapy: A Multi-Institutional Study
Source: Front Oncol. 2021 Nov 9;11:725507. doi: 10.3389/fonc.2021.725507 (PMC8630628; doi:10.3389/fonc.2021.725507)
Supplement: Supplementary file 1 [file DataSheet_1.docx]

Supplementary Material

**1 Supplementary Data**

The code are available and described in the following data record: <https://doi.org/10.6084/m9.figshare.14684412>.

**2 Supplementary Figures**


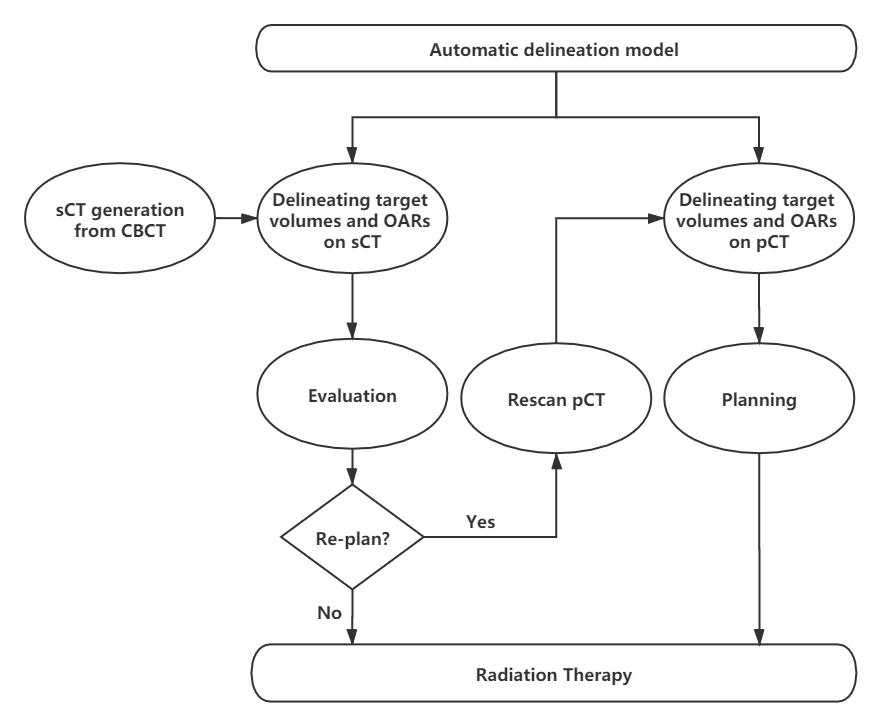


**Supplementary Figure 1.** The Flowchart of the proposed method for adaptive radiotherapy of breast cancer.

The sCT image was first generated from CBCT images with a cycle generative adversarial network (cycleGAN). Second, we developed an automatic delineation model using 3D U-net based on pCT and the radiotherapy structure of breast cancer patients to delineate the target volumes and OARs on pCT and sCT images, respectively. Third, the treatment plan was transferred to the sCT image from the pCT image. It could be verified quantitatively by quick dose recalculation for dosimetric evaluation, relying on gamma analysis and dose-volume histogram parameters. The clinical impact of geometric variations in target volumes and OARs was evaluated to decide whether to it was necessary to replan for breast cancer adaptive radiotherapy.


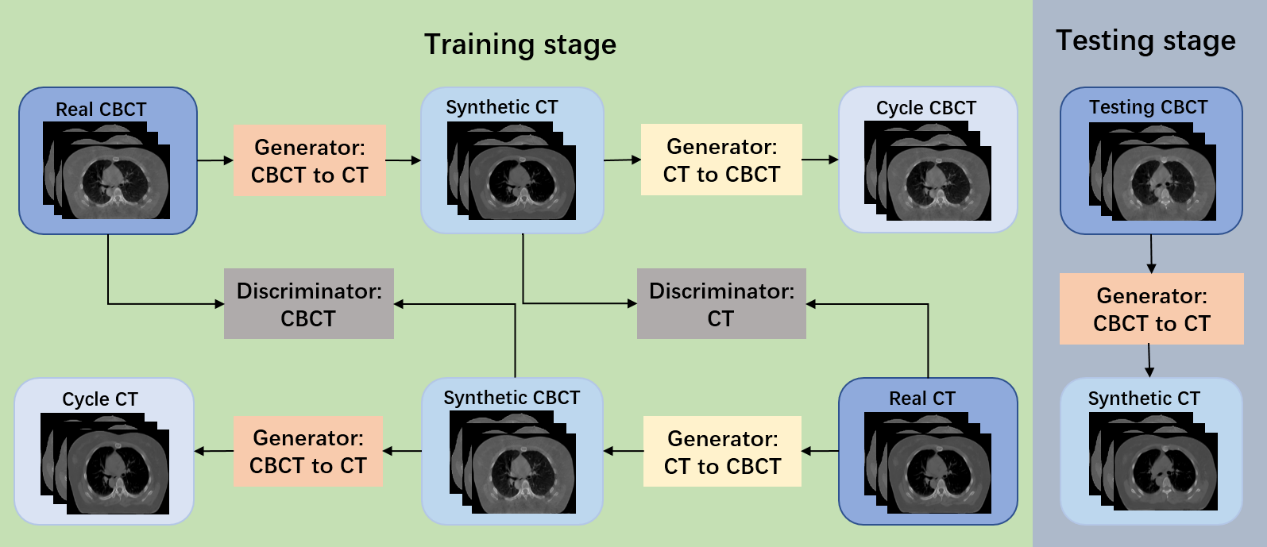


**Supplementary Figure 2.** The schematic flow of sCT generation with cycleGAN

The cycleGAN contains two generators and two discriminators. The generator takes CBCT as input and generates the sCT; in contrast, Generator CT to CBCT takes CT as input and generates the synthetic CBCT. The discriminator CT and CBCT discriminate whether the CT or CBCT images are real or synthesized, respectively.


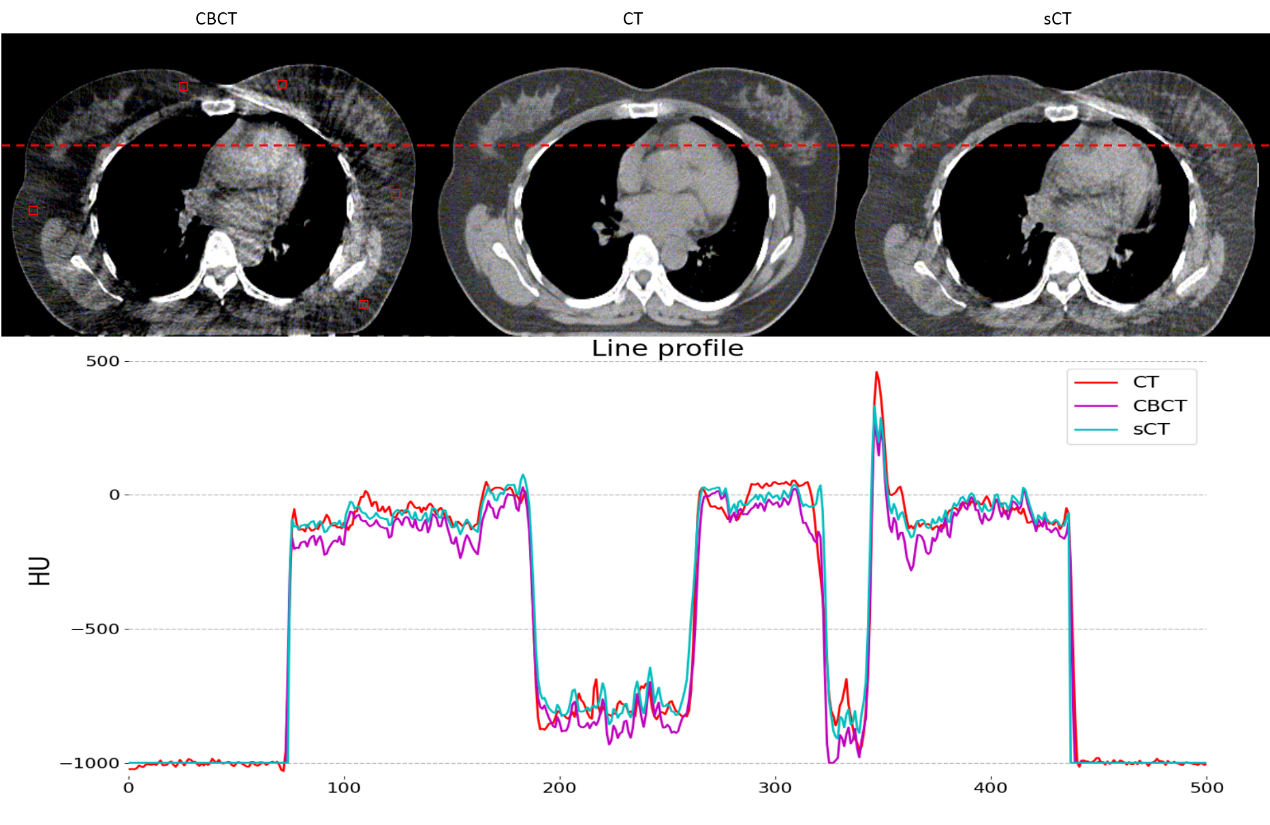


**Supplementary Figure 3.** Example showing HU line profiles of Cone-beam computed tomography (CBCT), synthetic CT(sCT), and reference CT.

The up row shows an example of CBCT, sCT, and pCT images of a test patient. The down plot shows HU profile of the dashed line in the up image. The red squares placed on the axial view of the image were selected ROIs for the SNU calculation. The HU value of sCT is much closer to that of pCT than that of CBCT. The CycleGAN reduced the artifacts of CBCT and increased the similarity with pCT.

**3** **Supplementary** **Equations**

Let X denote the ground-truth, let Y denote the automated delineation volume. DSC describes the spatial overlap between the automated delineation and the ground-truth. It is defined as follows:

$DSC=\frac{2\left|X\cap Y\right|}{\left|X\right|+\left|Y\right|}$ (1)

The metrics HD95 was used to evaluate the shape difference in the study. HD95 is the 95% percentile HD, which measures the 95^th^ percentile distance of all distances between points in X and the nearest point in Y. Let $X_{s}$ denote the boundary-surface set of the ground truth, let $Y_{s}$ denote the automated delineation.

HD is defined as:

$HD\left(X_{s},Y_{s}\right)=\max\left(d_{X_{s}Y_{s}},d_{Y_{s}X_{s}}\right)$ (2)

$d_{X_{S}Y_{S}}=\max\left(\min_{x\in X_{S},y\in Y_{S}} \left\|x-y\right\|\right)$ (3)

where $\left\|x-y\right\|$ indicates the Euclidean distance between voxels $x$ and $y$. $d_{X_{S}Y_{S}}$ describes the point $x\in X_{S}$ that is farthest from any point of $Y_{S}$ and calculates the distance from $x$ to its nearest neighbor in $Y_{S}$.

$ME=\frac{1}{N}\sum_{i}^{N} \left(sCT\left(v_{i}\right)-CT\left(v_{i}\right)\right)$ (4)

$MAE=\frac{1}{N}\sum_{i}^{N} \left|sCT\left(v_{i}\right)-CT\left(v_{i}\right)\right|$ (5)

$PSNR=10\times\log_{10}\left(\frac{MAX^{2}}{\sum_{i}^{N} \left|CT\left(v_{i}\right)-sCT\left(v_{i}\right)\right|^{2}/N}\right)$ (6)

$\mathrm{SSIM}(CT,sCT)=\frac{\left(2\mu_{CT}\mu_{sCT}+c_{1}\right)\left(2\sigma_{CT,sCT}+c_{2}\right)}{\left(\mu_{CT}^{2}+\mu_{sCT}^{2}+c_{1}\right)\left(\sigma_{CT}^{2}+\sigma_{sCT}^{2}+c_{2}\right)}$ (7)

$SNU=\frac{\bar{HU}_{max}-\bar{HU}_{min}}{1000}\times100\%$ (8)

where $sCT\left(v_{i}\right)$ is the HU value of the i-th pixel of sCT and $CT\left(v_{i}\right)$ is the i-th pixel of the CT in HU with N denoting the number of voxels within the body. MAX is the maximum intensity in CT and sCT. $\mu_{CT}$ and $\mu_{sCT}$ are the mean of CT and sCT image. $\sigma_{CT}$ and $\sigma_{sCT}$ are the standard deviations of CT and sCT images. $\bar{HU}_{max}$ and $\bar{HU}_{min}$ are the maximum and the minimum of the mean CT number values of regions of interest (ROIs), respectively.
